# Biases in the Detection of Intentionally Poisoned Animals: Public Health and Conservation Implications from a Field Experiment

**DOI:** 10.3390/ijerph18031201

**Published:** 2021-01-29

**Authors:** José M. Gil-Sánchez, Natividad Aguilera-Alcalá, Marcos Moleón, Esther Sebastián-González, Antoni Margalida, Zebensui Morales-Reyes, Carlos J. Durá-Alemañ, Pilar Oliva-Vidal, Juan M. Pérez-García, José A. Sánchez-Zapata

**Affiliations:** 1 Department of Applied Biology, Miguel Hernández University, Avda. de la Universidad, s/n, E-03202 Elche, Spain; jmgilsanchez@yahoo.es (J.M.G.-S.); esebgo@gmail.com (E.S.-G.); zmorales@umh.es (Z.M.-R.); jperez@umh.es (J.M.P.-G.); toni@umh.es (J.A.S.-Z.); 2 Department of Zoology, University of Granada, Avda. de Fuente Nueva, s/n, 18071 Granada, Spain; mmoleonpaiz@hotmail.com; 3 Institute for Game and Wildlife Research, IREC (CSIC-UCLM-JCCM), E-13005 Ciudad Real, Spain; a.margalida@csic.es (A.M.); pilar_olivavidal@hotmail.com (P.O.-V.); 4 International Center for Environmental Law Studies, CIEDA-CIEMAT, Bernardo Robles Square 9, 42002 Soria, Spain; cjavierdura@gmail.com; 5 Department of Animal Science (Division of Wildlife), Faculty of Life Sciences and Engineering, University of Lleida, E-25198 Lleida, Spain

**Keywords:** human-wildlife conflict, predator control, public health, vultures, wildlife conservation, wildlife poisoning

## Abstract

Intentional poisoning is a global wildlife problem and an overlooked risk factor for public health. Managing poisoning requires unbiased and high-quality data through wildlife monitoring protocols, which are largely lacking. We herein evaluated the biases associated with current monitoring programmes of wildlife poisoning in Spain. We compared the national poisoning database for the 1990–2015 period with information obtained from a field experiment during which we used camera-traps to detect the species that consumed non-poisoned baits. Our findings suggest that the detection rate of poisoned animals is species-dependent: Several animal groups (e.g., domestic mammalian carnivores and vultures) tended to be over-represented in the poisoning national database, while others (e.g., corvids and small mammals) were underrepresented. As revealed by the GLMM analyses, the probability of a given species being overrepresented was higher for heaviest, aerial, and cryptic species. In conclusion, we found that monitoring poisoned fauna based on heterogeneous sources may produce important biases in detection rates; thus, such information should be used with caution by managers and policy-makers. Our findings may guide to future search efforts aimed to reach a more comprehensive understanding of the intentional wildlife poisoning problem.

## 1. Introduction

Intentional poisoning is a critical global wildlife conservation problem that may affect individuals, populations, and even entire communities [1,2,3,4,5]. Consequently, many countries have passed strict legislation with severe penalties and invested considerable resources to reduce wildlife poisoning [1,3,6,7,8]. However, these measures have largely proved insufficient to date, and deliberate poisoning is still pervasive (e.g., [8,9]). Although many reasons lie behind this illegal activity, most are associated with human–wildlife conflicts related to predator control in game hunting and livestock farming areas [2,10], hence the most impacted species are mammalian carnivores and large raptors [2,6,11,12]. One dramatic example is the thousands of vultures that have been poisoned by elephant and rhino poachers and traditional medicine users in Africa in recent years [3,9,13].

Managing poisoning events requires high-quality data on the species, location and the number of individuals affected, which can be obtained through detection protocols of poisoned wildlife. However, as systematic approaches are rare, searching efforts may be biased by not only substantially underrepresenting the number of real events and affected individuals, but also overestimating the representation of certain species that may be more conspicuous or attractive to researchers and conservationists [3]. Frequently, poisoned animals are opportunistically detected by people who accidentally find them dead and inform local authorities. Thus, an accurate quantification of the potential impact of poison and its population-scale consequences requires improving the detection rate of poisoning animals and a comprehensive identification of affected species [3,14,15].

To date, several methods have been proposed to obtain a more complete approach to the wildlife poisoning problem. For instance, telemetry is a useful tool to evaluate the relative importance in the study population of different mortality causes, including poisoning [16,17]. However, telemetry is costly in both logistic and economic terms and its use is prevented on a large spatial-temporal scale. Dogs may also be efficiently trained to detect wildlife poisoning [18]. Currently, these canine patrols are successfully operating in Spain and other European countries, frequently as part of LIFE projects [19]. However, the information obtained through these procedures is probably biased towards the most likely detected species, such as the largest ones, domestic animals or endangered species, which are the object of monitoring programmes. To improve management and conservation actions, these biases should be evaluated.

Here we take Spain as a case study because it is one of the most biodiverse countries in the European Union according to the IUCN (https://www.iucn.org/regions/europe/resources/country-focus/spain), with 27.4% of the Spanish territory included in the Natura 2000 network. In this country, the use of poison has been considered a crime since 1995 (Organic Law 10/1995 of the Criminal Code), and at least 160 judgements have been made for illegal poisoning. National regulatory legislation was established in 2007 (Law 42/2007 on Natural Heritage and Biodiversity) and is applicable regionally by each Spanish Autonomous Community in different action plans. Yet despite Spain being a pioneer in the application of criminal law and administrative sanctions in Europe [20], wildlife poisoning is still a frequent illegal activity performed mainly to kill predators in game hunting areas and on pasturelands [2,18,21]. Every year, hundreds of individuals of threatened species, such as the Spanish imperial eagle (*Aquila adalberti*) [22], Egyptian vultures (*Neophron percnopterus*) [12], cinereous vultures (*Aegypius monachus*) [11], bearded vultures (*Gypaetus barbatus*) [16], and brown bears (*Ursus arctos*) [23], are poisoned. Furthermore, poisoning has been described as a major factor that contribute to the population decline of Egyptian vultures [24,25] and red kites (*Milvus milvus*) [4] on local and national scales. In addition, poisoning frequently involves domestic animals, including pets (particularly dogs and cats) and livestock [18,26], which might pose public health a risk. Given the environmental and public health consequences of intentional poisoning, research is necessary to improve the assessment of the risk associated with this activity.

Our main goal was to explore whether an experiment using non-poisoned baits could reveal the potential biases associated with the long-term Spanish poisoned fauna database. We compared this database to the information obtained from a field experiment in which we used camera-traps to detect the species that consumed non-poisoned baits. Our starting hypothesis was that the detection rate of poisoned animals would be species-dependent, which could lead to under or overestimate the representation of certain species in the poisoning database. Thus, our study could have profound implications for wildlife management, with important ramifications for domestic animals and human health.

## 2. Materials and Methods

### 2.1. Study Areas

**Figure 1:**
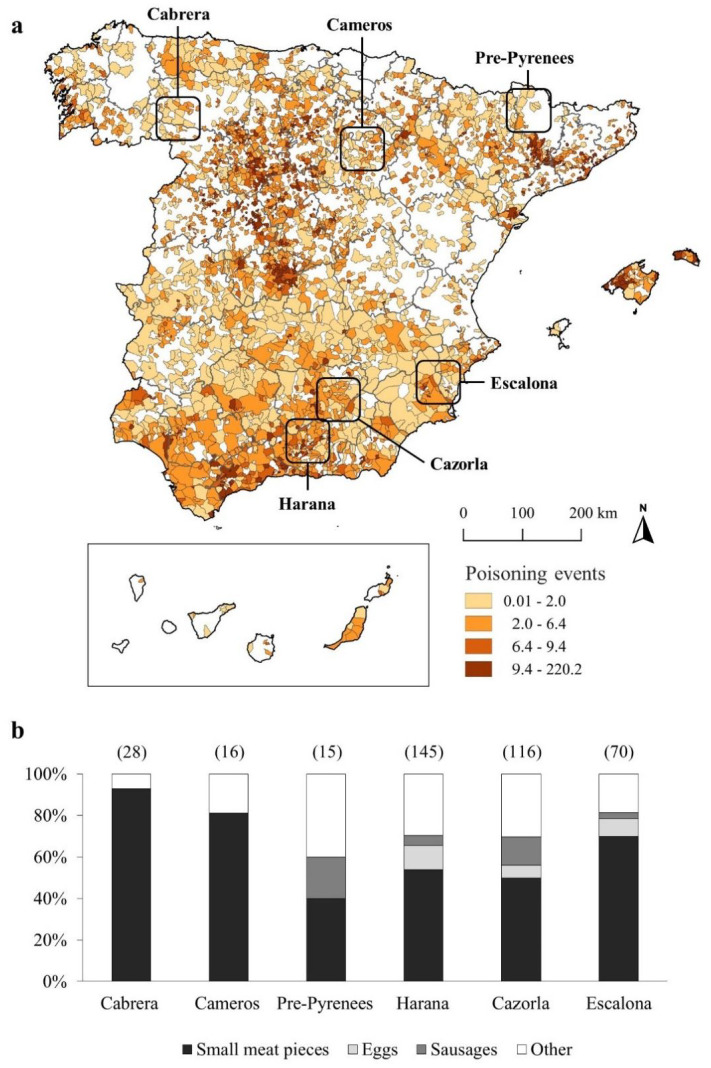
Poisoning events from the national database (“Antídoto Programme”) for the 1990–2015 period. (**a**) Distribution of poisoning events in Spain based on municipalities (no. of poisoning events per 100 km^2^ of municipality surface) and location of the six study areas. Grey lines delimit the administrative provinces. Blank areas denote lack of data, which should not be interpreted as zero poison. (**b**) Bait types used to poison the fauna in the study areas, as recorded in the national poisoning database. Number of events are in brackets.

The study was carried out in six areas of peninsular Spain (Figure 1a): Sierra de Cabrera (hereafter Cabrera); Sabinar de Calatañazor—Sierra de Cameros (hereafter Cameros); Pre-Pyrenees of Lleida (hereafter Pre-Pyrenees); Sierra Harana (hereafter Harana); Cazorla, Segura y Las Villas Natural Park (hereafter Cazorla); and Sierra Escalona (hereafter Escalona). These areas are located in different administrative regions (six provinces) and covering a wide gradient in environmental conditions (three Mediterranean areas and three transitional Mediterranean to Euro-Siberian areas), land uses (livestock types and densities, and intensity and type of sport game activities), presence and abundance of obligate scavengers and top predators (i.e., wolves *Canis lupus*), and degrees of protection, ranging from unprotected areas to the largest Spanish protected area (Cazorla, Figure 1a, Table 1). Thus, we obtained a wide representation of the socio-environmental variability that characterises peninsular Spain, including areas with the presence of particularly vulnerable species to poisoning (vultures) and others considered controversial that are usual targets for poisoning (wolves; [2]; see Table 1 for details).

### 2.2. Poisoning Database

We used the “Antídoto Programme” database (https://www.venenono.org/), which compiles data from poisoning events in Spain from 1990 to 2015. A poisoning event in this database is defined as the finding of a poisoned bait and/or a group of animals (one individual or mores of one species or several) poisoned by the same poison and bait types, and in a given spatiotemporal location (within 1 km and 1 month) [2,27]. For each event, data include the affected species, date and location. In all, the “Antídoto Programme” registered 18,212 poisoned individuals of 182 species, with widespread events throughout Spain (Figure 1a). Data availability varied depending on the administrative region, with some regions investing lots of poisoning search efforts (e.g., Andalusia, South Spain, Catalonia, Northeastern Spain), while others barely reported any data [28]. The most frequently found species were dogs (*Canis lupus familiaris*), red foxes (*Vulpes vulpes*), domestic cats (*Felis silvestris catus*), and several scavenging bird species [18]. Information on bait type was provided in 16.2% of database records (n = 2011), with the most commonly used being pieces of meat (50.7%), followed by sausages (6.5%) and eggs (5.7%). Bait usage frequency varied among areas (Figure 1b).

The location of poisoning events was recorded at the municipality level (Figure 1a). For our study, we extracted the number of poisoned individuals per species recorded in the municipalities included in each study area. Because of the limited sample size, we were unable to analyse the effects of bait type on the number of individuals and species affected by poisoning. This limitation also led us to consider the whole period for the available data without separating it into different subperiods.

### 2.3. Field Experiment

To detect and quantify the community of animal species that can potentially feed on poisoned baits, we designed a field experiment by a non-invasive methodology based on the monitoring of non-poisoned baits. As baits, we used those identified by the “Antídoto Programme” as the most widely used bait types: small meat pieces (one fresh chicken piece, 100 g), sausages (one sausage, 50 g) and eggs (three chicken eggs, 70 g each, placed together to simulate a ground nests). At each study site, we deployed 10 baits of each type from March 2019 to September 2019, which included the period of the year with the highest poisoning incidence in Spain [18]. Finally, we monitored the consumption process of 161 baits by means of passive infrared triggered cameras (Bushnell HD^TM^ and Browning Strike Force HD^TM^) placed 4–6 m away from baits. Cameras were programmed to take three pictures per trigger, with a 30-s delay whenever they detected movement. Meat pieces and sausages were fixed to the ground by wire or some similar material to ensure they were consumed in front of the cameras. Baits were placed on wildlife paths to simulate poisoners’ *modus operandi*. The distance between cameras was generally >1 km [29], except for Escalona and Pre-Pyrenees, where they were separated <200 m to simulate gamekeepers and shepherds’ reported local poisoning strategy. Experiments were reviewed weekly until bait had completely disappeared. If after 1 month bait remained untouched, we still finished the experiment. Generally, due to its small size, each bait was consumed only by one individual of each scavenger species that visited the site with bait. Thus, per individual bait, each consumer species was taken as one individual in the subsequent analyses.

### 2.4. Ethic Statement

We conducted the field experiment without handling or disturbing wildlife. Authorisations to photograph wildlife using bait attractants were obtained from the respective administrative offices in the study areas (EP/SO/389/2019, SF/002, 47/CV/20, SGMN/GyB/JMIF). If the study was conducted on private property, express authorisation was requested from owners.

### 2.5. Statistical Analyses

First, we classified the different species into nine groups following taxonomic and behavioural criteria as follows: wild carnivores (i.e., mammalian carnivores); domestic carnivores (dogs and cats); suids (wild boar *Sus scrofa*); small mammals (rodents, shrews, hedgehogs); corvids; vultures; other raptors; other birds; and reptiles. Separately for each study area, we compared the representation of each group (frequency of occurrence) between both data sources: the “Antídoto Programme” poisoned animals database and the field experiment results. Frequency of occurrence was defined as the percentage of the total number of individuals: (a) poisoned (the “Antídoto Programme” database) and (b) recorded consuming baits (field experiment) that corresponded to a given group. Comparisons of frequencies were made by χ^2^ tests on 2 × 2 contingency tables. Bonferroni’s correction was applied for *P*-levels.

Second, we used Generalised Linear Mixed Models (GLMMs; [30]) to explore the factors that potentially influenced the differences between both data sources (the “Antídoto Programme” database and the field experiment) in the representation of the different species. Specifically for a given species in a certain area, our response variable was the number of poisoned individuals included in the “Antídoto Programme” database, minus the number of baits consumed by that species during the field experiment. Thus, the value could be positive or negative, depending on whether the species was over- or underrepresented, respectively, in the poisoning database in relation to the field experiment data. Study area was included as random factor, while fixed factors were: species weight (mean adult—female and male—weight in Spain in kg; log-transformed); colour (conspicuous –with the presence of black and white or shiny black patches—/cryptic otherwise); mobility (aerial—birds—/terrestrial—mammals and reptiles—); sociality (social—foraging in large or family groups—/solitary—foraging alone or in pairs—); and conservation status (endangered/non-endangered) (see Table S1 for species details). The weight data were obtained from the Virtual Encyclopaedia of Spanish Vertebrates (http://www.vertebradosibericos.org/) and [31]. First, we constructed two models with all the explanatory variables (no interactions were considered given the small sample size): one with no random term and another one with a random term. Then we selected the model with the most appropriate random structure by a restricted maximum likelihood (REML) procedure and the glmer() function of the *lme4* package of R [32]. We used Gaussian error distributions and identity link functions. Having identified the most suitable random structure (i.e., with a random term; see the Results), we selected the model with the most appropriate fixed structure using maximum likelihood (ML). For this purpose, we explored the complete set of alternative models using the dredge() function of the *MuMIn* package of R [33]. We then proceeded to model selection, according to Akaike’s information criterion corrected for small sample sizes (AIC_c_). By this approach, we identified the most parsimonious model (lowest AIC_c_) by ranking the remaining models from the lowest to highest delta-AIC_c_ (the difference in AIC_c_ between each model and the most parsimonious model). We considered those models with delta-AIC_c_ < 2 to have similar support [34]. Then we recalculated the selected model by REML, and the resulting model was taken as the final model. The final model’s performance was assessed by calculating marginal *R*^2^, which measures how much variability of the response variable is explained by the model’s fixed term [35]. To do so, we used the r.squaredGLMM() function of the *MuMIn* package of R [33]. All the analyses were conducted with the R statistical software (https://www.r-project.org/).

## 3. Results

**Figure 2:**
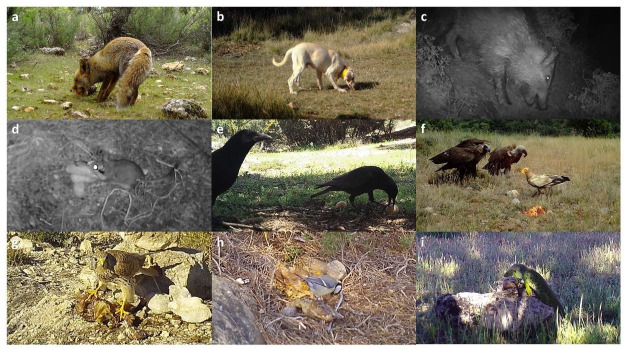
Individuals of the nine species groups feeding on experimental baits. (**a**). Wild carnivores, *Vulpes vulpes*. (**b**). Domestic carnivores, *Canis lupus familiaris*. (**c**). Suids, *Sus scrofa*. (**d**). Small mammals, *Eliomys* *quercinus*. (**e**). Corvids, *Corvus corone*. (**f**). Vultures, *Aegypius monachus*, *Gyps fulvus* and *Neophron percnopterus*. (**g**). Other raptors, *Falco tinnunculus*. (**h**). Other birds, *Parus major*. (**i**). Reptiles, *Timon lepidus*.

**Figure 3:**
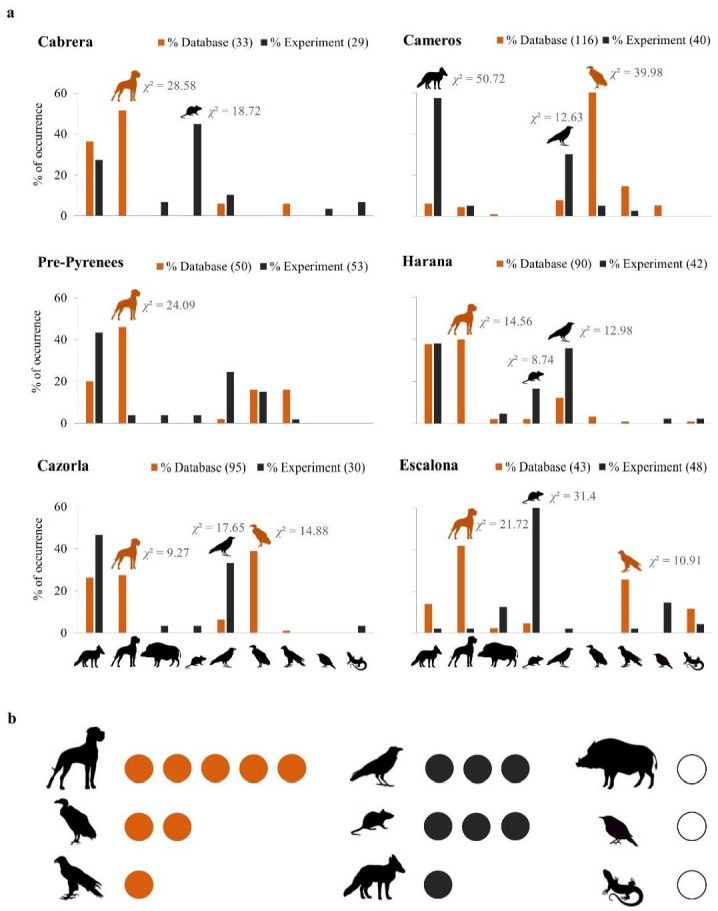
Comparison between the national poisoned fauna database (“Antídoto Programme”) and the field experiment results according to the different species groups and study areas. (**a**) Frequency of occurrence of individuals in each group recorded in the “Antídoto Programme” (orange bars) compared to the percentage of baits consumed during the field experiment per group (from left to right: wild carnivores, domestic carnivores, suids, small mammals, corvids, vultures, other raptors, other birds, and reptiles). Brackets indicate the total number of poisoned individuals in the database and the estimated total number of different individuals that consumed baits (see the text for details). The pair-wise χ^2^ tests results are shown whenever we detected significant differences between both data sources (*p* < 0.05). (**b**) Number of study areas in which each group was over- (orange dots) or underrepresented (black dots) in the national database compared to the results of experimental baits. White dots denote the groups for which we detected no significant differences between data sources.

In the six study areas, we detected 38 species actually or potentially suffering from intentional poisoning (27 species included in the poisoning database and 26 species feeding on the experimental baits) (Table 2 and Table 3, Figure 2). According to the poisoning database, the most frequent groups were wild and domestic carnivores, vultures, and other raptors, while wild carnivores, small mammals and corvids were the groups that most frequently consumed experimental baits (Figure 3b). The most represented species in the study areas were domestic dogs (108 individuals poisoned; three experimental baits consumed), griffon vultures (104 individuals poisoned; seven experimental baits consumed) and red foxes (62 individuals poisoned; 57 experimental baits consumed; Table S2). The pool of species detected either in the field experiment or the poisoning database well represents the Spanish community of wild carnivores, corvids and raptors, especially vultures, as the four European species were represented between the two data sources (Table 2). Most of the chicken pieces (95.8%) and sausages (95.1%) used in the experiment were completely consumed, while nearly half the egg groups (41.2%) remained untouched at the end of the experiment.

We found differences (*p* < 0.0005 with Bonferroni’s correction) between both data sources for 15 comparisons (27.7% of 54 χ^2^ comparisons), with three groups detected at higher rates than expected by random in eight comparisons (14.8%) and another three groups were underdetected in seven comparisons (12.9%; Figure 3). In general, the results among the six areas were consistent: For wild carnivores and other raptors, we found no differences between data sources in five areas; for domestic carnivores, we found an over-representation in the poisoning database in five areas; for suids, other birds and reptiles, of which very few were detected, we found no differences between data sources in all the areas (Figure 3). Corvids and small mammals were underrepresented in the poisoning database in three areas, and vultures were overrepresented in this database in two areas; in other areas, we found no difference in the representation of these groups between data sources (Figure 3). Thus, we found no contrasting results for any group; i.e., overrepresentation in the poisoning database in one area, but underrepresentation in the poisoning database in another area, or vice versa.

As revealed by the GLMMs (Table S3), the factors that influenced the differences between the number of poisoned individuals included in the “Antídoto Programme” database and the number of individuals recorded consuming baits during the field experiment were the species’ weight, colour and mobility. According to marginal *R*^2^, this model explained c. 20% of the variability in the response variable. Study area (i.e., random term) was also included in the selected model (Table S3). However, this variable explained very little variability of the response variable (0.7%), which agrees with the results described in the previous paragraph. In particular, heavy, aerial and cryptic species were overrepresented in the poisoning database vs. the field experiment compared to light, terrestrial and conspicuous species (Table 3).

## 4. Discussion

By a field experiment to identify the community of species that are potentially poisoned in different Spanish areas, we evaluated possible biases in the detection rates of poisoned animals resulting from current wildlife poisoning monitoring programmes to offer a pioneering approach to this key global wildlife conservation problem [36].

Our main hypothesis was supported by data as the detection rates differed among poisoned species. We identified three factors that influence the probability of a species being over- or underrepresented in the poisoning database in relation to bait-based field experiment results. First, larger species were more susceptible to be overrepresented in the poisoning database, which is likely because these species are easier to detect by searchers [37]. Second, compared to mammals and reptiles, birds were more frequently poisoned than expected according to the field experiment, which could be explained by secondary poisoning. Birds like vultures and other raptors might feed on the carcasses of animals that have been previously poisoned. Secondary poisoning may affect mainly birds because of their higher response capacity due to great searching and movement abilities compared to terrestrial animals [38,39]. Our experiment was unable to capture these cascading effects, which could be relevant to fully assess the impact of poisoning along the food web, as previously described for rodenticides [40]. Third, conspicuous species like those herein defined (see Materials and Methods) were more susceptible to be underrepresented in the poisoning database. This is probably because most conspicuous species were either rare (e.g., bearded and Egyptian vultures), with a limited contribution to the global pattern, or, especially, corvids. Corvids comprise a relatively conspicuous group of medium-sized birds with non-cryptic colours that are relatively abundant in the study areas. Thus, their underrepresentation in poisoning records could be related to a lesser local effort in monitoring them as they are not target species of conservation programmes. The fact that they are common could imply that they are ignored when considering species affected by poisoning events. However, this should not be the case of regions in which efficient canine patrols operate, specifically if they look for poisoned baits and dead animals. Moreover, we assigned only one individual to each species that was observed feeding on a given bait. This could lead to an underrepresentation of small gregarious species (e.g., several corvid species) as larger species like carnivores normally ate all the bait. Therefore, alternative explanations for low corvid detection rates by poisoned fauna searchers are required, but difficult to establish.

Besides corvids, poisoned small mammals and wild carnivores were underrepresented in poisoning records. This was expected for small mammals because this group includes several small-sized burrowing species, which are difficult to detect after poisoning events [41], especially in closed habitats. In addition, small carcasses could also degrade faster [42] and may disappear before being detected. Also, several small mammal species are considered “pests”, which may lead searchers to ignore them. To solve this problem, it is necessary to develop specific monitoring programmes for those scenarios with endangered small-sized species. Despite being the subject of several scientific and conservation monitoring programmes in Spain, the representation of mammalian carnivores in the poisoning database was no higher than that expected based on the field experiment results. Indeed, poisoned wild carnivores were underrepresented in one study area (Cameros), a result that could be partially related to local monitoring procedures, which apparently focus more on locating poisoned vultures and partially disregard other poisoned species (Figure 3). In any case, other factors like species-specific behavioural responses might influence these results. Carnivores, like corvids, are major facultative scavengers worldwide [43,44] and may display flexible behaviours and outstanding learning capacity [45,46]. These abilities can help them to avoid consuming poisoned baits and other risky carrion sources [29]. Thus, with fierce poisoning pressure, carnivores and corvids might refuse to eat potentially risky baits. For example, our experiment found that wolves detected at least two (non-poisoned) baits in the only study areas where they were present, but did not consume them.

Poisoned pets like dogs and cats, were recorded in the national poisoning database more often than when they were observed in the field experiment in most of the study areas. This was an expected result because these pets are easier to detect than smaller species. In addition, a higher presence of pets would be expected near inhabited areas [47], whereas field experiments were run in natural areas, usually far from these areas. Moreover, dogs and cats are probably more familiar with the bait types commonly used for poisoning than wild species are [48], which could increase the risk of the former being poisoned due to reduced aversion or neophobia to baits [49]. Also, pet owners might often report the occurrence of poison to authorities because they look for their missing pets or contact their veterinarians whenever they suspect a poisoning event [26]. Pets might pose a high public health risk because of their proximity to humans. Similarly, poisoned wild boars could also be taken into account because of their potential consumption by humans.

We found some geographical variations in the detection bias of poisoned fauna. Geography-based variations were affected by administrative divisions, which determined: (1) the local poison sources and preferred way to poison (Figure 1b); and (2) the different poisoning monitoring approaches followed by environmental authorities [18]. For example, the specialised canine patrols that detect poisoned animals or baits in some Spanish Autonomous Communities (e.g., Andalusia, where two of the study areas were located: Harana and Cazorla) are well-known for increasing the efficiency of searches [18]. In addition, the presence and management of hunting areas may affect poisoning type and intensity [11,12]. Unfortunately, the “Antídoto Programme” database does not allow a more detailed analysis of the different geographical scenarios because it does not record the methodological approaches used to detect poisoning. In any case, the fact that the study area was relatively unimportant in the GLMMs indicated that geographical differences must be related mainly to the detection rate of poisoned animals (i.e., with some areas invest more search efforts than others) rather than to differences in the probability of over- or underrepresenting poisoned species. This suggests that the people involved in poisoning detection from different administration may share similar search criteria. However, whether these criteria for searching poisoned fauna have changed with time is an open question.

### Conclusions and Conservation Implications

Intentional poisoning is a global conservation concern for scavenger species, especially vultures [3,13,24,50,51], but also other endangered species [4]. Poisoning big game species, such as wild boars, and pet species, such as dogs and cats, may pose a risk for humans. Thus, deliberate poisoning is not only a wildlife problem, but an environmental and human health matter.

We found potentially important biases in the detection of different animal groups that are vulnerable to deliberate poisoning, even within administrative divisions with exceptional global poisoning monitoring programmes. We particularly highlight that the detection rate of poisoned fauna from current monitoring schemes based on heterogeneous sources is strongly species-dependent. So, these data must be interpreted with caution. Having identified the several species traits influencing the detection rate of poisoned animals, our findings may guide future search efforts to more comprehensively understand the extent of the intentional poisoning problem. This could mean that, from a practitioner viewpoint, the personnel responsible for wildlife vigilance (e.g., guards, rangers, gamekeepers) can conform to an appropriate tool to detect poisoning events that affect vultures and other raptors, especially if they are supported by local people previously trained by education and engagement programmes [18]. Given the biases revealed by this field experiment, more attention should be paid to consider small mammals and corvids found dead as susceptible to poisoning. Few administrative regions currently operate with specialised canine units, which are essential to improve the detection of poisoned fauna [37]. Therefore, implementing canine units in all regions would be a priority in the fight against wildlife poisoning [52]. All these measures could benefit from the establishment of a national body that coordinates anti-poisoning strategies [52]. Only by developing unbiased monitoring programmes for deliberate poisoning can we achieve healthy ecosystems for wildlife, domestic animals and humans.

## Figures and Tables

**Table 1:** Description of the six study areas.

Area	Habitat Type	Hunting Type/Intensity	Natural Park	Livestock/Density	Vulture Abundance	Wolf Abundance
Cabrera	Mosaic of transitional Mediterranean to Euro-Siberian scrubs, forests and meadows	Small and big game/low	No	Cattle, sheep/high	Low	High
Pre-Pyrenees	Mosaic of transitional Mediterranean to Euro-Siberian forests and meadows	Small and big game/low	No	Cattle, sheep/high	High	(Absent)
Cameros	Mosaic of transitional Mediterranean to Euro-Siberian forests and meadows	Small and big game/medium	No	Cattle, horses, sheep/high	High	Low
Harana	Mosaic of xeric Mediterranean scrubs, forests and crops	Small and big game/high	No	Sheep, goats/low	Low	(Absent)
Cazorla	Mediterranean forests	Big game/medium	Yes	Sheep, goats/medium	High	(Absent)
Escalona	Mosaic of xeric Mediterranean scrubs, forests and crops	Small game/high	No	Sheep/very low	(Absent)	(Absent)

**Table 2:** Number of species recorded as poisoned in the “Antídoto Programme” database and observed feeding on baits during the field experiment in the six study areas per species group. The total shows the number of species that occurred in both the database and the experiment. We also show the number of species of each group that are present in peninsular Spain, along with the percentage of species that represents those involved in this study.

Source	Wild Carnivores	Domestic Carnivores	Suids	Small Mammals	Corvids	Vultures	Other Raptors	Other Birds	Reptiles	N
Database	6	2	1	2	4	3	9	1	1	27
Experiment	5	2	1	3	5	3	3	4	2	26
Total	7		1	5	5	4	9	5	2	38
Spain	16 (44%)		1 (100%)	34 (15%)	9 (56%)	4 (100%)	31 (29%)	528 (1%)	57 (4%)	680 (6%)

**Table 3:** Selected Linear Generalised Mixed Model (GLMM) showing the variables explaining the difference between (a) the number of poisoned individuals included in the “Antídoto Programme” database and (b) the number of individuals recorded consuming baits during the field experiment. The estimates of the parameters (including the sign) and the standard error of parameters (SE) are shown.

Parameter	Estimate	SE
(Intercept)	1.154	1.325
weight	3.612	0.950
color (conspicuous)	−5.139	1.960
mobility (aerial)	4.289	1.863

## Data Availability

The data obtained in the field experiment of this study are available in the Supplementary Material. Data from “Antídoto Programme” should be requested to WWF Spain and SEO/BirdLife.

## Supplementary Materials

The following are available online at https://www.mdpi.com/1660-4601/18/3/1201/s1, Table S1: Species traits considered in the GLMM. We included species weight (mean adult weight in Spain, in kg), color (conspicuous—with presence of black and white or bright black patches—/cryptic—otherwise—), mobility (aerial—birds—/terrestrial—mammals and reptiles—), sociality (social—foraging in large groups or familiar groups—/solitary—foraging alone or in pairs—), and conservation status (endangered/non-endangered), Table S2: Species and individuals recorded in the “Antídoto Program” database (“Dat.”) and observed feeding upon baits in the field experiment (“Exp.”), by study area. Species considered as endangered according to Spanish national and/or regional laws are indicated by an asterisk, Table S3. AICc-based model selection to assess the effect of study area (random factor) and weight, color, mobility, sociality, and conservation status of the species on the difference between a) the number of poisoned individuals included in the “Antídoto Program” database and b) the number of individuals recorded consuming the baits in the field experiment. Number of estimated parameters (k), AICc values, AICc differences (delta-AICc) with the highest ranked model (i.e., the one with the lowest AICc), and the variability of the response variable that is explained by the fixed factors (R2) are shown. The selected model is in bold.

## References

[1] Guitart R., Sachana M., Caloni F., Croubels S., Vandenbroucke V., Berny P. (2010). Animal poisoning in Europe. Part 3: Wildlife. Vet. J..

[2] Mateo-Tomás P., Olea P.P., Sánchez-Barbudo I.S., Mateo R. (2012). Alleviating human-wildlife conflicts: Identifying the causes and mapping the risk of illegal poisoning of wild fauna. J. Appl. Ecol..

[3] Ogada D.L. (2014). The power of poison: Pesticide poisoning of Africa’s wildlife. Ann. N. Y. Acad. Sci..

[4] Mateo-Tomás P., Olea P.P., Mínguez E., Mateo R., Viñuela J. (2020). Direct evidence of poison-driven widespread population decline in a wild vertebrate. Proc. Natl. Acad. Sci. USA.

[5] Di Blasio A., Bertolini S., Gili M., Avolio R., Leogrande M., Ostorero F., Ru G., Dondo A., Zoppi S. (2020). Local context and environment as risk factors for acute poisoning in animals in northwest Italy. Sci. Total. Environ..

[6] Berny P., Gaillet J.R. (2008). Acute poisoning of Red Kites (*Milvus milvus*) in France: Data from the SAGIR network. J. Wildl. Dis..

[7] Margalida A. (2012). Baits, budget cuts: A deadly mix. Science.

[8] Margalida A., Mateo R. (2019). Illegal killing of birds in Europe continues. Science.

[9] Ogada D., Botha A., Shaw P. (2016). Ivory poachers and poison: Drivers of Africa’s declining vulture populations. Oryx.

[10] Lozano J., Olszańska A., Morales-Reyes Z., Castro A.A., Malo A.F., Moleón M., Sánchez-Zapata J.A., Cortés-Avizanda A., von Wehrden H., Dorresteijn I. (2019). Human-carnivore relations: A systematic review. Biol. Conserv..

[11] Hernández M., Margalida A. (2008). Pesticide abuse in Europe: Effects on the Cinereous vulture (*Aegypius monachus*) population in Spain. Ecotoxicology.

[12] Hernández M., Margalida A. (2009). Poison-related mortality effects in the endangered Egyptian vulture (*Neophron percnopterus*) population in Spain. Eur. J. Wildl. Res..

[13] Margalida A., Ogada D., Botha A. (2019). Protect African vultures from poison. Science.

[14] Vyas N.B. (1999). Factors influencing estimation of pesticide-related wildlife mortality. Toxicol. Ind. Health.

[15] Mineau P., Ralph C.J., Rich T.D. (2005). Direct losses of birds to pesticides—Beginnings of a quantification. Bird Conservation Implementation and Integration in the Americas: Proceedings of the Third International Partners in Flight Conference, Asilomar, CA, USA, 20–24 March 2002.

[16] Margalida A., Heredia R., Razin M., Hernández M. (2008). Sources of variation in mortality of the Bearded Vulture *Gypaetus barbatus* in Europe. Bird Conserv. Int..

[17] Treves A., Artelle K.A., Darimont C.T., Parsons D.R. (2017). Mismeasured mortality: Correcting estimates of wolf poaching in the United States. J. Mammal..

[18] De la Bodega D., Cano C., Mínguez E., SEO/BirdLife, WWF (2020). El Veneno en España. Evolución del Envenenamiento de Fauna Silvestre (1992–2017).

[19] Silva J.P., Eldridge J., Nottinghan S., Travagnin C. (2018). Life & Wildlife Crime.

[20] Durá-Alemañ C.J., Morales-Reyes Z., Ayerza P., De la Bodega D., Aguilera-Alcalá N., Botella F., Jiménez-Peinado J., Jiménez J., López-Bao J.V., Mateo-Tomás P. (2020). La responsabilidad por el daño ambiental generado en el caso de la lucha contra el uso del veneno en España. Actual. Jurid. Ambient..

[21] Cano C., De la Bodega D., Ayerza P., Mínguez E. (2016). El Veneno en España. Evolución del Envenenamiento de Fauna Silvestre (1992–2013).

[22] González L.M., Oria J., Sánchez R., Margalida A., Aranda A., Prada L., Caldera J., Molina J.I. (2008). Status and habitat changes in the endangered Spanish Imperial Eagle *Aquila adalberti* population during 1974–2004: Implications for its recovery. Bird Conserv. Int..

[23] Naves J., Wiegand T., Fernández-Gil A., Stephan T. (1999). Riesgo de Extinción del oso Pardo Cantábrico. La Población Occidental.

[24] Carrete M., Grande J.M., Tella J.L., Sánchez-Zapata J.A., Donázar J.A., Díaz-Delgado R., Romo A. (2007). Habitat, human pressure, and social behavior: Partialling out factors affecting large-scale territory extinction in an endangered vulture. Biol. Conserv..

[25] Sanz-Aguilar A., Sánchez-Zapata J.A., Carrete M., Benítez J.R., Ávila E., Arenas R., Donázar J.A. (2015). Action on multiple fronts, illegal poisoning and wind farm planning, is required to reverse the decline of the Egyptian vulture in southern Spain. Biol. Conserv..

[26] Berny P., Caloni F., Croubels S., Sachana M., Vandenbroucke V., Davanzo F., Guitart R. (2010). Animal poisoning in Europe. Part 2: Companion animals. Vet. J..

[27] Whitfield D.P., McLeod D.R.A., Watson J., Fielding A.H., Haworth P.F. (2003). The association of grouse moor in Scotland with the illegal use of poisons to control predators. Biol. Conserv..

[28] Cano C. (2017). La Lucha Contra el Veneno en España (2011–2016). Clasificación por Comunidades Autónomas.

[29] Moleón M., Martínez-Carrasco C., Muellerklein O.C., Getz W.M., Muñoz-Lozano C., Sánchez-Zapata J.A. (2017). Carnivore carcasses are avoided by carnivores. J. Anim. Ecol..

[30] Zuur A., Leno E.N., Walker N., Saveliev A.A., Smith G.M. (2009). Mixed Effects Models and Extensions in Ecology with R.

[31] Blanco J.C. (1998). Mamíferos de España. Vol. I and II.

[32] Bates D., Maechler M., Bolker B., Walker S. (2013). lme4: Linear Mixed-Effects Models Using Eigen and S4. R Package Version 1.0–5.

[33] Barton K. (2013). MuMIn: Multi-Model Interference. R Package Version 1.9.13.

[34] Burnham K.P., Anderson D.R. (2002). Model Selection and Multimodel Inference. A Practical Information-Theoretic Approach.

[35] Nakagawa S., Schielzeth H. (2013). A general and simple method for obtaining R2 from generalized linear mixed-effects models. Methods Ecol. Evol..

[36] Richards N. (2012). Carbofuran and Wildlife Poisoning: Global Perspectives and Forensic Approaches.

[37] Barrientos R., Martins R.C., Ascensão F., D’Amico M., Moreira F., Borda-de-Água L. (2018). A review of searcher efficiency and carcass persistence in infrastructure-driven mortality assessment studies. Biol. Conserv..

[38] Ruxton G.D., Houston D.C. (2004). Obligate vertebrate scavengers must be large soaring fliers. J. Theor. Biol..

[39] Gutiérrez-Cánovas C., Moleón M., Mateo-Tomás P., Olea P.P., Sebastián-González E., Sánchez-Zapata J.A. (2020). Large home range scavengers support higher rates of carcass removal. bioRxiv.

[40] Brakes C.R., Smith R.H. (2005). Exposure of non-target small mammals to rodenticides: Short-term effects, recovery and implications for secondary poisoning. J. Appl. Ecol..

[41] Moleón M., Selva N., Quaggiotto M.M., Bailey D.M., Cortés-Avizanda A., DeVault T.L., Olea P.P., Mateo-Tomás P., Sánchez-Zapata J.A. (2019). Carrion availability in space and time. Carrion Ecology and Management.

[42] Moleón M., Sánchez-Zapata J.A., Sebastián-González E., Owen-Smith N. (2015). Carcass size shapes the structure and functioning of an African scavenging assemblage. Oikos.

[43] Mateo-Tomás P., Olea P.P., Moleón M., Vicente J., Botella F., Selva N., Viñuela J., Sánchez-Zapata J.A. (2015). From regional to global patterns in vertebrate scavenger communities subsidized by big game hunting. Divers. Distrib..

[44] Sebastián-González E., Barbosa J.M., Pérez-García J.M., Morales-Reyes Z., Botella F., Olea P.P., Mateo-Tomás P., Moleón M., Hiraldo F., Arrondo E. (2019). Scavenging in the Anthropocene: Human impact drives vertebrate scavenger species richness at a global scale. Glob. Chang. Biol..

[45] Emery N.J., Clayton N.S. (2004). The mentality of crows: Convergent evolution of intelligence in corvids and apes. Science.

[46] Moe R.O., Bakken M., Kittilsen S., Kingsley-Smith H., Spruijt B.M. (2006). A note on reward-related behaviour and emotional expressions in farmed silver foxes (*Vulpes vulpes*)-Basis for a novel tool to study animal welfare. Appl. Anim. Behav. Sci..

[47] Thomas R.L., Baker P.J., Fellowes M.D.E. (2014). Ranging characteristics of the domestic cat (*Felis catus*) in an urban environment. Urban Ecosyst..

[48] Jarjour C., Evans J.C., Routh M., Morand-Ferron J. (2020). Does city life reduce neophobia? A study on wild black-capped chickadees. Behav. Ecol..

[49] Bradshaw J.W.S. (2006). The evolutionary basis for the feeding behavior of domestic dogs (*Canis familiaris*) and cats (*Felis catus*). J. Nutr..

[50] Berny P., Vilagines L., Cugnasse J.M., Mastain O., Chollet J.Y., Joncour G., Razin M. (2015). Vigilance Poison: Illegal poisoning and lead intoxication are the main factors affecting avian scavenger survival in the Pyrenees (France). Ecotoxicol. Environ. Saf..

[51] Ogada D.L., Keesing F., Virani M.Z. (2012). Dropping dead: Causes and consequences of vulture population declines worldwide. Ann. N. Y. Acad. Sci..

[52] Durá-Alemañ C.J., Ayerza P., Cano C., Jiménez J., Sánchez-Zapata J.A., Morales-Reyes Z. (2021). Las sentencias contra el veneno en España. Quercus.

