# Supplementary material for: Biases in the Detection of Intentionally Poisoned Animals: Public Health and Conservation Implications from a Field Experiment
Source: Int J Environ Res Public Health. 2021 Jan 29;18(3):1201. doi: 10.3390/ijerph18031201 (PMC7908198; doi:10.3390/ijerph18031201)
Supplement: Supplementary file 1 [file ijerph-18-01201-s001.pdf]

Supplementary Material for

Biases in the detection of intentionally poisoned animals: public health and conservation implications from a field experiment

José M. Gil-Sánchez, Natividad Aguilera-Alcalá, Marcos Moleón, Esther Sebastián-González, Antoni Margalida, Zebensui Morales-Reyes, Carlos J. Durá Alemañ, Pilar Oliva-Vidal, Juan M. Pérez-García and José A. Sánchez-Zapata

This material includes:

Table S1. Species traits considered in the GLMM.
Table S2. Species and individuals recorded in the "Antídoto Program" database and observed feeding upon baits in the field experiment, by study area.
Table S3. AIC_c-based model selection.

Table S1. Species traits considered in the GLMM. We included species weight (mean adult weight in Spain, in kg), color (conspicuous – with presence of black and white or bright black patches – / cryptic – otherwise –), mobility (aerial – birds – / terrestrial – mammals and reptiles –), sociality (social – foraging in large groups or familiar groups – / solitary – foraging alone or in pairs –), and conservation status (endangered / non-endangered).

Group	Species	Weight (kg)	Log weight	Color	Mobility	Sociality	Conservation status
Wild carnivores	*Canis lupus*	30	1.48	cryptic	terrestrial	social	endangered
	Felis silvestris silvestris	4	0.60	cryptic	terrestrial	solitary	endangered
	Genetta genetta	1.9	0.28	conspicuous	terrestrial	solitary	non-endangered
	Martes foina	1.75	0.24	cryptic	terrestrial	solitary	non-endangered
	Martes martes	1.75	0.24	cryptic	terrestrial	solitary	non-endangered
	Meles meles	7.5	0.88	conspicuous	terrestrial	social	non-endangered
	Vulpes vulpes	6	0.78	cryptic	terrestrial	solitary	non-endangered
Domestic carnivores	*Canis lupus familiaris*	15	1.18	cryptic	terrestrial	solitary	non-endangered
	Felis silvestris catus	4	0.60	cryptic	terrestrial	solitary	non-endangered
Suids	*Sus scrofa*	52.98	1.72	cryptic	terrestrial	social	non-endangered
Small mammals	*Apodemus sylvaticus*	0.025	-1.60	cryptic	terrestrial	solitary	non-endangered
	Crocidura russula	0.008	-2.10	cryptic	terrestrial	solitary	non-endangered
	Eliomys quercinus	0.09	-1.05	conspicuous	terrestrial	solitary	non-endangered
	Erinaceus europaeus	0.8	-0.10	cryptic	terrestrial	solitary	non-endangered
	Rattus spp.	0.3	-0.52	cryptic	terrestrial	solitary	non-endangered
Corvids	*Corvus corax*	2	0.30	conspicuous	aerial	social	non-endangered
	Corvus corone	0.6	-0.22	conspicuous	aerial	social	non-endangered
	Cyanopica cooki	0.07	-1.15	conspicuous	aerial	social	non-endangered
	Garrulus glandarius	0.19	-0.72	conspicuous	aerial	solitary	non-endangered
	Pica pica	0.24	-0.62	conspicuous	aerial	social	non-endangered
Vultures	*Aegypius monachus*	9.8	0.99	conspicuous	aerial	social	endangered
	Gypaetus barbatus	6	0.78	conspicuous	aerial	solitary	endangered

	Gyps fulvus	8.5	0.93	cryptic	aerial	social	non-endangered
	Neophron percnopterus	2	0.30	conspicuous	aerial	solitary	endangered
Other raptors	*Accipiter gentilis*	1	0.00	cryptic	aerial	solitary	non-endangered
	Aquila chrysaetos	4	0.60	cryptic	aerial	solitary	endangered
	Aquila fasciata	2.2	0.34	cryptic	aerial	solitary	endangered
	Bubo bubo	1.75	0.24	cryptic	aerial	solitary	non-endangered
	Buteo buteo	0.73	-0.14	cryptic	aerial	solitary	non-endangered
	Falco tinnunculus	0.225	-0.65	cryptic	aerial	solitary	non-endangered
	Hieraaetus pennatus	0.8	-0.10	cryptic	aerial	solitary	non-endangered
	Milvus migrans	0.75	-0.12	cryptic	aerial	social	non-endangered
	Milvus milvus	1	0.00	cryptic	aerial	social	endangered
Other birds	*Ciconia ciconia*	3.5	0.54	conspicuous	aerial	social	non-endangered
	Erithacus rubecula	0.019	-1.72	cryptic	aerial	solitary	non-endangered
	Parus major	0.018	-1.74	conspicuous	aerial	solitary	non-endangered
	Phoenicurus ochruros	0.016	-1.80	cryptic	aerial	solitary	non-endangered
	Turdus viscivorus	0.13	-0.89	cryptic	aerial	solitary	non-endangered
Reptiles	*Timon lepidus*	0.25	-0.60	cryptic	terrestrial	solitary	non-endangered
	Lacerta schreiberi	0.026	-1.59	cryptic	terrestrial	solitary	endangered

Table S2. Species and individuals recorded in the "Antídoto Program" database ("Dat.") and observed feeding upon baits in the field experiment ("Exp."), by study area. Species considered as endangered according to Spanish national and/or regional laws are indicated by an asterisk.

		**Cabrera**		**Cameros**		**Pre-Pyrenees**		**Harana**		**Cazorla**		**Escalona**		**Total**	
**Group**	**Species**	**Dat.**	**Exp.**	**Dat.**	**Exp.**	**Dat.**	**Exp.**	**Dat.**	**Exp.**	**Dat.**	**Exp.**	**Dat.**	**Exp.**	**Dat.**	**Exp.**
Wild carnivores	*Canis lupus**	9	0	0	0	0	0	0	0	0	0	0	0	**9**	**0**
	*Felis silvestris silvestris**	0	0	0	0	0	0	0	0	1	0	0	0	**1**	**0**
	Genetta genetta	0	0	0	0	1	0	0	0	0	1	0	0	**1**	**1**
	Martes foina	0	0	0	9	6	6	10	2	2	6	0	0	**18**	**23**
	Martes martes	0	1	0	0	0	0	0	0	0	0	0	0	**0**	**1**
	Meles meles	0	0	0	1	1	1	2	1	0	0	0	0	**3**	**3**
	Vulpes vulpes	3	7	7	13	2	16	22	13	22	10	6	1	**62**	**59**
Domestic carnivores	*Canis lupus familiaris*	17	0	5	1	20	2	26	0	23	0	17	0	**108**	**3**
	Felis silvestris catus	0	0	0	1	3	0	10	0	3	0	1	1	**17**	**2**
Suids	*Sus scrofa*	0	2	1	0	0	2	2	2	0	1	1	6	**4**	**13**
Small mammals	*Apodemus sylvaticus*	0	9	0	0	0	2	1	4	0	1	2	3	**3**	**19**
	Crocidura russula	0	0	0	0	0	0	1	0	0	0	0	0	**1**	**0**
	Eliomys quercinus	0	3	0	0	0	0	0	3	0	0	0	25	**0**	**31**
	Erinaceus europaeus	0	1	0	0	0	0	0	0	0	0	0	0	**0**	**1**
	Rattus spp.	0	0	0	0	0	0	0	0	0	0	0	1	**0**	**1**
Corvids	*Corvus corax*	0	0	9	5	1	6	1	0	4	0	0	0	**15**	**11**
	Corvus corone	0	0	0	3	0	0	0	4	2	5	0	0	**2**	**12**
	Cyanopica cooki	0	0	0	0	0	0	1	2	0	0	0	0	**1**	**2**
	Garrulus glandarius	0	3	0	4	0	7	0	5	0	5	0	0	**0**	**24**
	Pica pica	2	0	0	0	0	0	9	4	0	0	0	1	**11**	**5**
Vultures	*Aegypius monachus**	0	0	0	0	0	1	0	0	0	0	0	0	**0**	**1**
	*Gypaetus barbatus**	0	0	0	0	1	0	0	0	4	0	0	0	**5**	**0**
	Gyps fulvus	0	0	64	2	7	5	3	0	30	0	0	0	**104**	**7**
	*Neophron percnopterus**	0	0	7	0	0	4	0	0	3	0	0	0	**10**	**4**
Other raptors	*Accipiter gentilis*	0	0	0	0	1	0	1	0	0	0	1	0	**3**	**0**
	*Aquila chrysaetos**	0	0	1	0	0	0	0	0	0	0	0	0	**1**	**0**
	*Aquila fasciata**	0	0	0	0	0	0	0	0	0	0	5	0	**5**	**0**
	Bubo bubo	0	0	1	0	0	0	0	0	0	0	2	0	**3**	**0**
	Buteo buteo	0	0	4	0	2	0	0	0	0	0	2	0	**8**	**0**
	Falco tinnunculus	0	0	0	0	0	0	0	0	1	0	1	1	**2**	**1**
	Hieraaetus pennatus	0	0	1	0	0	0	0	0	0	0	0	0	**1**	**0**

	Milvus migrans	0	0	0	1	1	0	0	0	0	0	0	0	**1**	**1**
	*Milvus milvus**	2	0	10	0	4	1	0	0	0	0	0	0	**16**	**1**
Other birds	*Ciconia ciconia*	0	0	6	0	0	0	0	0	0	0	0	0	**6**	**0**
	Erithacus rubecula	0	0	0	0	0	0	0	0	0	0	0	2	**0**	**2**
	Parus major	0	0	0	0	0	0	0	1	0	0	0	4	**0**	**5**
	Phoenicurus ochruros	0	0	0	0	0	0	0	0	0	0	0	1	**0**	**1**
	Turdus viscivorus	0	1	0	0	0	0	0	0	0	0	0	0	**0**	**1**
Reptiles	*Lacerta schreiberi**	0	2	0	0	0	0	0	0	0	0	0	0	**0**	**2**
	Timon lepidus	0	0	0	0	0	0	1	1	0	1	5	2	**6**	**4**
**Total**		**33**	**29**	**116**	**40**	**50**	**53**	**90**	**42**	**95**	**30**	**43**	**48**	**427**	**237**

Table S3. AIC_c-based model selection to assess the effect of study area (random factor) and weight, color, mobility, sociality, and conservation status of the species on the difference between a) the number of poisoned individuals included in the "Antídoto Program" database and b) the number of individuals recorded consuming the baits in the field experiment. Number of estimated parameters (k), AIC_c values, AIC_c differences (delta-AIC_c) with the highest ranked model (i.e., the one with the lowest AIC_c), and the variability of the response variable that is explained by the fixed factors (R^2) are shown. The selected model is in bold.

**Model**	**k**	**AIC_c**	**delta-AIC_c**	**marginal R^2**
**weight + color + mobility (+ 1|area)**	**4**	**721.6**	**0**	**19.69**
weight + color + mobility + status (+ 1|area)	5	723.8	2.21	
weight + color + mobility + sociality (+ 1|area)	5	723.9	2.28	
weight + color (+ 1|area)	3	724.5	2.88	
weight (+ 1|area)	2	725.9	4.31	
weight + mobility (+ 1|area)	3	726.0	4.38	
weight + color + sociality (+ 1|area)	4	726.1	4.50	
weight + color + mobility + sociality + status (+ 1|area)	6	726.2	4.55	
weight + color + status (+ 1|area)	4	726.7	5.09	
weight + mobility + sociality (+ 1|area)	4	728.1	6.51	
weight + mobility + status (+ 1|area)	4	728.1	6.51	
weight + sociality (+ 1|area)	3	728.1	6.51	
weight + status (+ 1|area)	3	728.2	6.52	
weight + color + sociality + status (+ 1|area)	5	728.4	6.73	
weight + mobility + sociality + status (+ 1|area)	5	730.2	8.57	
weight + sociality + status (+ 1|area)	4	730.4	8.77	
color + sociality (+ 1|area)	3	731.7	10.04	
color + mobility + sociality (+ 1|area)	4	732.5	10.89	
color + mobility (+ 1|area)	3	733.4	11.81	
color + sociality + status (+ 1|area)	4	733.5	11.88	
color (+ 1|area)	2	733.6	11.95	
color + mobility + sociality + status (+ 1|area)	5	734.7	13.03	
color + status (+ 1|area)	3	735.3	13.71	
color + mobility + status (+ 1|area)	4	735.6	13.94	
(1|area)	1	737.2	15.53	
sociality (+ 1|area)	2	737.7	16.04	
mobility (+ 1|area)	2	739.1	17.50	
status (+ 1|area)	2	739.1	17.50	
sociality + status (+ 1|area)	3	739.7	18.08	
mobility + sociality (+ 1|area)	3	739.8	18.21	
mobility + status (+ 1|area)	3	741.2	19.53	
mobility + sociality + status (+ 1|area)	4	742.0	20.33	
